# iTRAQ-Based Quantitative Proteomics Analysis on Rice Anther Responding to High Temperature

**DOI:** 10.3390/ijms18091811

**Published:** 2017-08-23

**Authors:** Qilin Mu, Wenying Zhang, Yunbo Zhang, Haoliang Yan, Ke Liu, Tsutomu Matsui, Xiaohai Tian, Pingfang Yang

**Affiliations:** 1 Agricultural College, Yangtze University, Jingzhou 434025, China; mql325@163.com (Q.M.); wyzhang@yangtzeu.edu.cn (W.Z.); yunbo1022@126.com (Y.Z.); yanhl1989@163.com (H.Y.); keliu928@126.com (K.L.); 2 Hubei Collaborative Innovation Center for Grain Industry, Jingzhou 434025, China; 3 Applied Biological Faculty, Gifu University, Gifu 501-1193, Japan; matsuit@gifu-u.ac.jp; 4 Key Laboratory of Plant Germplasm Enhancement and Speciality Agriculture, Wuhan Botanical Garden, Chinese Academy of Sciences, Wuhan 430074, China

**Keywords:** rice, high temperature, iTRAQ, proteomics, spikelet fertility

## Abstract

As one of the most important crops, rice provides the major food for more than half of the world population. However, its production is limited by many environmental factors, among which high temperature stress (HS) frequently occurs during anthesis and reduces its spikelet fertility. To explore the mechanism of HS tolerance in rice, we conducted a comparative proteomics analysis on the anthers between HS resistant and sensitive cultivars under different levels of high temperature. Under the same HS treatment, the resistant cultivar showed much higher spikelet fertility than the sensitive cultivar. Proteomic data showed that HS lead to the degradation of ribosomal proteins in the sensitive cultivar but not in the resistant one, which might result in the injury of protein biosynthetic machinery. In contrast, HS induced the increase of sHSP, β-expansins and lipid transfer proteins in the resistant cultivar, which might contribute to its ability to tolerate HS. The results provide some new insights into the mechanism of rice HS response.

## 1. Introduction

With the increasing of world population, it has been a challenge to provide enough and stable food supply. Among all the crops, rice might be the most important one being the major food for more than half of the world population, especially in Asia [1]. During production, rice has to face different abiotic stresses, which negatively affect its growth and productivity. Because of their sessile characters, field grown plants have evolved diverse strategies to combat abiotic stresses depending on ecology, timing, severity and the stage of growth to improve survival [2]. With the industrialization of the world, global warming has become a threat to food production, which might lead to more than 25% decrease of the major food yield by 2050, as predicted by the International Panel on Climate Change [3]. To secure the rice supply, it is critical to develop rice cultivar with not only high yield, but also great stress tolerance.

In the last 50 years, high temperature has been one of the serious threatens for rice production. It has been reported that a 1 °C increase in temperature can lead to 7–8% of reduction in rice yield [4]. Although nearly all growth stages of the whole life cycle of rice are negatively affected by high temperature [5], anthesis is believed to be the most sensitive one [6,7]. There are over 2 × 10^7^ ha of rice cultivation in the area along Yangtze River, which makes it one of the largest rice production areas. However, high temperature usually happens from the booting to flowering stages during rice growth, especially in the middle and down-stream area of Yangtze River [8,9]. It has been known that high temperature can reduce the fertility of rice spikelet [10]. Actually, many different physiological processes during the anthesis, including anther dehiscence, pollination and pollen germination, and growth are negatively influenced by heat stress (HS) [11,12,13,14]. However, one hour after pollination, HS no longer affects fertility [6].

It is one of the major goals for rice breeder to develop HS resistant cultivar [15,16]. It has been suggested that rice resistance to HS is a quantitative trait [17]. Quantitative traits loci (QTLs) explaining 12.6% and 17.6% of the total HS tolerance variation have been detected on chromosome 4 (qHTSF4.1) and chromosome 1 (qHTSF1.1) in rice cultivar N22, respectively [18]. N22 is known to be the most HS tolerant variety up-to-date [19], which showed much less yield decrease under HS [12,17]. Very few rice cultivars are as tolerant as N22 to HS, which indicates the existence of molecular mechanisms contributing its tolerance to HS. Different techniques have been applied to study the rice HS response, including microarray [20], transcriptomics [21] and proteomics [17,22]. miRNA in responding to HS were also profiled [23]. However, these studies were conducted on either sensitive or tolerant cultivar alone. Very few studies were conducted in comparison of the sensitive and tolerant cultivars. 

Very recently, Gonzalez et al. [24] conducted a genome-wide transcriptome analysis in both tolerant and sensitive varieties. In addition, Kim et al. [14] applied gel-based proteomics study in three rice varieties, including HS high sensitive, middle tolerant and high tolerant one. However, because of the limitation of gel resolution, insufficient information was obtained. In the current study, we conducted an isobaric tags for relative and absolute quantitation (iTRAQ) -labeling-based quantitative proteomic study to compare the different HS responses between the tolerant cultivar N22 and the sensitive cultivar Mianhui101. Specifically, different degrees of HS were applied, which may help to obtain more comprehensive insights into the molecular mechanisms underlying rice HS response at anthesis stage.

## 2. Results and Discussion

### 2.1. High Temperature Tolerance of N22 and Mianhui101

**Figure 1:**
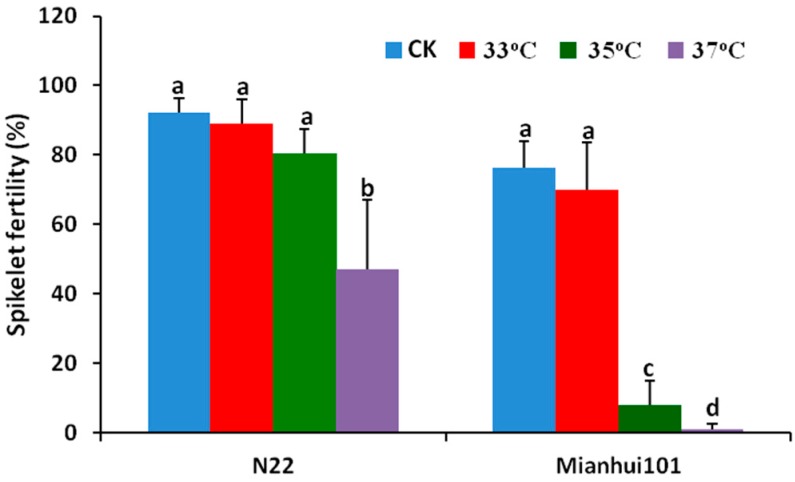
Quantitative analysis of the spikelet fertility in two rice cultivars under different high temperature stress. CK stands for the control treatment. The letters a, b, c and d stand for different levels of significance. Data are means ± SE (standard error) (*n* = 3, *p* < 0.05). One-way ANOVA analysis was performed.

It has been reported that N22 is a HS tolerant variety [19], and showed weaker HS response [12,17]. In contrast, Mianhui101 is more sensitive to HS. To verify their responses to HS, the spikelet fertility was measured for both cultivars under varied heat regimes at anthesis stage. Under control treatment (CK), the spikelet fertility for N22 and Mianhui101 were 92.1 ± 4.1% and 76.3 ± 7.6%, respectively (Figure 1). For the treatment with 33 °C, both cultivars showed little decrease on the fertility without significant difference. When HS increased to 35 °C, the fertility of N22 decreased to 83.2 ± 4.8%, whereas that of Mianhui101 dramatically decreased to 8.1 ± 6.8% (Figure 1). Under 37 °C, Mianhui101 was totally sterile, while N22 still had about 47.1% fertility (Figure 1). Previous study has shown that N22 could keep good fertility even under 38 °C treatment [12]. In summary, the fertility of N22 decreased much slower than that of Mianhui101 with the increase of high temperature, which verified that N22 is much more tolerant to HS than Mianhui101. Based these data, it is concluded that the two cultivars are suitable for further comparative study on their HS responsive mechanisms.

### 2.2. High Temperature Induced Proteome Changes in N22 and Mianhui101

**Figure 2:**
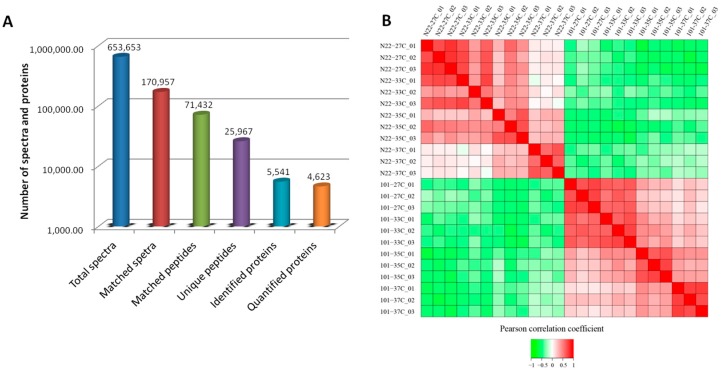
Results of mass spectrometry analysis and protein identification (**A**); and Pearson correlation analysis among the samples from different biological replicate (**B**). N22 and 101 stand for the two cultivars; 27C stands for the CK treatment; 33C, 35C and 37C stand for different HS treatments; and the digital number at the end of each sample indicates the biological repeat.

To further explore the molecular mechanisms that lead to the different responses to HS, iTRAQ-based proteomics strategy was applied to analyze the proteome changes in the anthers of both cultivars. After protein extraction, enzyme digestion and iTRAQ labeling, all samples were subjected to LC-MS/MS independently in three replicates. In total, 653,653 spectra were detected, among which 170,957 could be matched to 71,432 peptides with 25,967 being unique peptides (Figure 2A). Based on the criteria described in Materials and Methods, 5541 proteins were identified, of which 4623 were quantified (Figure 2A). The dataset is much larger than previous proteomic studies on the rice anther in responding to HS [12,14,25]. Pearson correlation analysis showed good correlation among different replicates of the same sample, whereas there are big differences between the two cultivars (Figure 2B). It could also be judged that 33 °C treatment seems to have no effect on the proteome profile of N22 since there are good correlations between this treated sample and the control (Figure 2B). 

**Figure 3:**
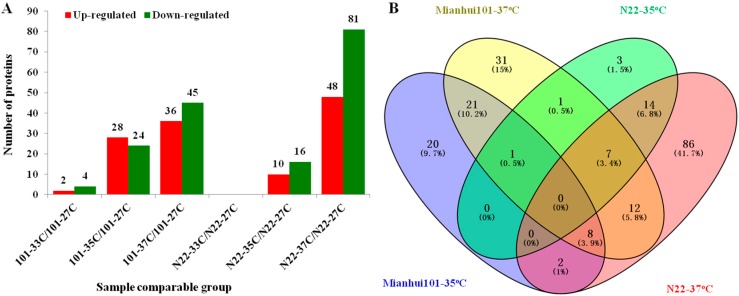
Quantitative analysis of the proteome between the HS treated and control samples (**A**); and Venn analysis among the samples from different HS treatment of two rice cultivars (**B**). N22 and 101 stand for the two cultivars; 27C stands for the CK treatment; and 33C, 35C and 37C stand for different HS treatment.

To identify the HS responsive proteins in both cultivars, proteins with more than 1.5-fold changes in abundance (*p* < 0.05) between the HS treatment and CK were selected as differentially expressed. Based on this criterion, there are six differentially expressed proteins, including two up-regulated and four down-regulated, in the sensitive cultivar Mianhui101, whereas there are no changed proteins in the tolerant cultivar N22 under 33 °C treatment (Figure 3A). The few or no changes on proteome at 33 °C treatment in both cultivars are consistent with the results on spikelet fertilities. Thus, we focused on the differentially expressed proteins under 35 and 37 °C treatments for further analysis. Under 35 °C treatment, there are 52 (28 up-regulated and 24 down-regulated) and 26 (10 up-regulated and 16 down-regulated) changed proteins for Mianhui101 and N22, respectively (Figure 3A). The two cultivars had just one protein in common at 35 °C (Figure 3B). When the temperature increased to 37 °C, the differentially expressed proteins in Mianhui101 and N22 were 81 (36 up-regulated and 45 down-regulated) and 129 (48 up-regulated and 81 down-regulated), respectively (Figure 3A). At 37 °C, the two cultivars had 27 proteins in common (Figure 3B). There were 21 proteins that could only be detected in Mianhui101 but not in N22 under both temperatures; on the contrary, there were 14 proteins only in N22 at both temperatures (Figure 3B).

### 2.3. Functional Categorization and Gene Ontology (GO) Analysis of the Changed Proteins

**Figure 4:**
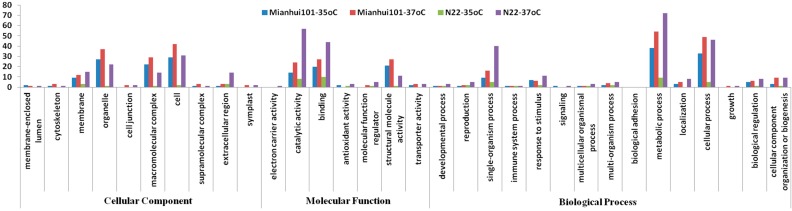
Gene ontology analysis of the differentially expressed proteins in both cultivars under 35 and 37 °C treatment. The *y*-axis stands for the number of proteins.

To further understand the function of the differentially expressed proteins, IDs of all the 4623 quantified proteins were searched against the UniProt-GOA database as described in Materials and Methods to obtain their function information (Table S2). Three categories, cellular compartment, biological process and molecular function by gene ontology (GO) annotation derived from UniProt-GOA database (www.http://www.ebi.ac.uk/GOA/) were assigned among all the differentially expressed proteins (Figure 3). Based on the GO analysis, the differentially expressed proteins belong to 15 biological processes, 10 cellular compartments, and seven different molecular functions. In terms of biological process, metabolic process, cellular process and single-organism process were the three major groups, which indicate that the primary metabolic processes are very easy to be affected in responding to HS. Actually, this is quite popular in plant exposed to different stresses [26]. Cell, organelle and macromolecular complex were the top three cellular compartments, which imply the general effects of HS on cell structure. Catalytic activity, binding and structural molecular activity were the three major molecular functional groups. Among most of the functional groups, N22 under 35 °C has the least number of proteins (Figure 4), which is consistent with the fact that its spikelet fertility was affected to the least level. This also indicates that HS could result in similar damages on both cultivars. Specifically, the numbers of proteins belonging to the immune system process group in biological process category were almost the same among the four samples (Figure 4), indicating the initiation of general responsive mechanisms under HS. Very obviously, the extracellular region containing proteins are much more abundant in the N22 under 37 °C than in any other samples (Figure 4). Among these proteins, there are two α-expansins, three β-expansins, one expansin-B13 and three lipid transfer proteins (Table S2). Expansin can non-enzymatically mediate a pH dependent cell wall loosening, thus enabling cell expansion [27]. It is also indicated that expansions might involve in the pollen tube growth and stigma surface loosening in sexual reproduction [27]. In this study, the two α-expansins were down-regulated, whereas the three β-expansins and expansin-B13 were up-regulated by the 37 °C HS in N22. This is consistent with previous report that α-expansins mainly function in eudicot cell walls but are less effective on grass cell walls, whereas β-expansins were more effective in grass pollen [28]. In contrast, both α-expansins and three β-expansins were down-regulated in Mianhui 101. The three lipid transfer proteins were also up-regulated (Table S2). Guo et al. [29] identified a similar drought-responsive lipid transfer protein OsDIL in rice anther, which could support the pollen fertility under different abiotic stresses. The increase of β-expansins and lipid transfer proteins might contribute to the resistance to HS in N22.

### 2.4. N22 and Mianhui101 Specifically High Temperature Stress (HS) Responsive Proteins

As mentioned above, there were 14 N22 and 21 Mianhui101 specifically differentially expressed proteins (Figure 3B). These cultivar specific but not temperature specific proteins might contribute to the different HS responses in the two varieties. 

Among the 14 N22-specific proteins, three were up-regulated and 11 were down-regulated under both temperatures (Table 1). All three up-regulated proteins were stress response proteins, with two small Heat shock proteins (sHSPs) and one peroxidase. The peroxidase is involved in cellular redox homeostasis. Under 35 °C treatment, there was one isoform of peroxidase up-regulated in N22, and another isoform up-regulated in Mianhui101. However, under 37 °C treatment, there were two isoforms of peroxidase increased in N22, but none were detected in Mianhui101 (Table S3). Although there were two HSPs detected as up-regulated under 37 °C treatment, none was detected under 35 °C treatment in Mianhui101 (Table S3). In contrast, there were four and seven HSPs detected as up-regulated in N22 under 35 and 37 °C treatments, respectively (Table S3). The different responsive expression of *HSPs* was also detected at the mRNA level in another study [24]. HSPs are very important factors that act as chaperone to help the functional proteins correctly fold under stresses [30]. In plant, there are five types of HSPs according to their molecular weight: HSP100, HSP90, HSP70, HSP60, and sHSPs [31]. The sHSPs can prevent irreversible unfolding or wrong protein aggregation through binding to the partially folded or denatured proteins [32], which might facilitate these proteins’ further refolding by Hsp70/Hsp100 complexes [33]. It seems that there is a positive qualitative relation between the accumulation of sHSPs in the plastids and thermotolerance of heat shock in the temperature range from 28 to 40 °C in plants [34]. It was also indicated that the mitochondrial sHSP could protect NADH: ubiquinone oxidoreductase (complex I) during heat stress [35]. Altogether, it might indicate some role of these sHSPs in tolerance of rice to HS.

All the down-regulated proteins were either continually decreased along with the increase of temperature or showing similar decrease at both temperatures. Among them, there are five related to metabolism, three to plant growth and development, and one each to cell wall, chloroplast and membrane (Table 1). Based on the features of these proteins, it could be generally concluded that HS had negative effect on this resistant cultivars. Specifically, an anther-specific protein RTS decreased almost half of its amount under 35 and 37 °C treatments. This RTS is required for male fertility [36]. Down-regulation of this gene could result in reducing male fertility in rice, which might explain the reduction of spikelet fertility in N22.

Among the 21 Mianhui101 specific proteins, 11 were up-regulated and 10 were down-regulated (Table 2). Interestingly, we did not detect the decrease of RTS in Mianhui101 under HS, which indicates that the reduction of spikelet fertility by HS might be mediated by a different pathway in Mianhui101 from that in N22. Nine of the 10 down-regulated proteins were ribosomal proteins, affiliating to either large or small subunit. Ribosome is the machinery for protein de-novo biosynthesis. Based on the proteomics data, it is obvious that the protein biosynthesis might be negatively affected by HS in Mianhui101, which should be one of the major reasons that lead to the sacrifice of spikelet fertility.

## 3. Materials and Methods

### 3.1. Rice Materials and Growth

Two *Oryza sativa indica* cultivars, named N22 and Mianhui101, were used in this study. The N22 is tolerant, while Mianhui101 is sensitive to high temperature. Seeds of both cultivars were germinated at room temperature at the second half of April. After 20 days, rice seedlings with similar height were transferred into plastic pots with the size of 30 cm in height and 30 cm of diameter, and then cultivated in the experimental farm of Yangtze University. For each pot, 12.5 kg of soil was mixed with 8 g composed fertilizer (with a ratio of 26:10:15 on N:P:K), and then filled with water constantly.

### 3.2. High Temperature Treatment and Sampling

Rice plants for both cultivars at anthesis stage were subjected to control and three different high temperature treatments for 3 days as follows. One day before anthesis, the rice plants were moved into growth chamber (Conviron Company, PGW40, Winnipeg, MB, Canada) to start the heat treatment with a 14-h-day/10-h-night cycle and a 2-h change on the temperature simulating the local typical heat stress weather. For control and high temperature treatments, the temperatures were set as in Table S1. The actual temperature regimes were about 27 (control), 30, 32 and 34 °C in daily mean temperature, respectively, and their corresponding daily maximum temperature were 31, 33, 35 and 37 °C. They are named as control, 33, 35 and 37 °C treatment, accordingly. The relative humidity was 70% and 80% in day and night, respectively. After about 72 h treatment, sampling was conducted at 15:00–16:00 on the spikelets estimated to open the next day. The anthers were pulled out and collected with specific vacuum aspiration equipment promptly and then stored in the iced dish. The samplers were brought back to the lab and kept at liquid nitrogen for protein extraction. The plants for fertility estimation were moved into the chamber with control temperature until all the spikelets were flowered, and moved to the open field until the seeds matured.

### 3.3. Protein Extraction, Digestion and iTRAQ Labeling

The proteins were extracted as previous reported with minor modifications [37]. Briefly, the anther sample was ground in liquid nitrogen, then the powder was transferred to 5 mL centrifuge tube and sonicated three times on ice using a high intensity ultrasonic processor (Scientz) in lysis buffer (8 M urea, 2 mM Ethylene Diamine Tetraacetic Acid (EDTA), 10 mM Dithiothreitol (DTT) and 1% Protease Inhibitor Cocktail). The debris was removed by centrifugation at 20,000× *g* at 4 °C for 10 min. Finally, the protein was precipitated with cold 15% Trichloroacetic acid (TCA) for 2 h at −20 °C. After centrifugation at 4 °C for 10 min, the supernatant was discarded. The remaining precipitant was washed with cold acetone for three times. The protein was redissolved in sample buffer (8 M urea, 100 mM Triethylamonium bicarbonat (TEAB), pH 8.0) and the protein concentration was determined with 2-dimensional (2-D) Quant kit according to the manufacturer’s instructions.

The in-solution digestion and iTRAQ Labeling were performed as previous reported [38]. Briefly, the protein was reduced with 10 mM DTT for 1 h at 37 °C and alkylated with 20 mM IAA for 45 min at room temperature in darkness. After diluted with 100 mM TEAB, trypsin was added into the protein sample at 1:50 trypsin-to-protein mass ratio and kept at 37 °C overnight. Approximately 100 μg protein for each sample was digested for the following experiments. After trypsin digestion, peptides were desalted by Strata X C18 SPE column (Phenomenex, Torrance, CA, USA) and vacuum-dried, and then reconstituted in 0.5 M TEAB and processed according to the manufacturer’s protocol for 8-plex iTRAQ kit. Mianhui101 at 37, 35, 33 °C and control treatments were labeled iTRAQ113, -114, -115 and -116, respectively; N22 at the corresponding temperatures were labeled iTRAQ117, -118, -119 and -121, respectively.

### 3.4. Quantitative Proteomic Analysis by LC-MS/MS and Protein Identification by Database Searching

Before mass spectrometry analysis, the sample was mixed and then fractionated into 18 fractions by high pH reverse-phase HPLC with a gradient of 2% to 60% acetonitrile (ACN, Fisher Chemical, Pittsburgh, PA, USA) in 10 mM ammonium bicarbonate pH 10 using Agilent 300Extend C18 column (5 μm particles, 4.6 mm ID, 250 mm length, Santa Clara, CA, USA).

Each fraction of peptides was vacuum dried, and then dissolved in 0.1% formic acid (FA, Fluka), directly loaded onto a reversed-phase pre-column (Acclaim PepMap 100, Thermo, Pittsburgh, PA, USA). Peptide separation was performed using a reversed-phase analytical column (Acclaim PepMap RSLC, Thermo). The gradient was comprised of an increase from 6% to 22% solvent B (0.1% FA in 98% ACN) over 26 min, 22% to 35% in 8 min and climbing to 80% in 3 min then holding at 80% for the last 3 min, all at a constant flow rate of 400 nL/min on an EASY-nLC 1000 UPLC system. The peptides were subjected to neutral spray ionization (NSI) source followed by tandem mass spectrometry (MS/MS) in Q Exactive^TM^ plus (Thermo) coupled online to the UPLC. Intact peptides were detected in the Orbitrap at a resolution of 70,000. Peptides were selected for MS/MS using NCE setting as 28, 32; ion fragments were detected in the Orbitrap at a resolution of 17,500. A data-dependent procedure that alternated between one MS scan followed by 20 MS/MS scans was applied for the top 20 precursor ions above a threshold ion count of 10,000 in the MS survey scan with 30.0 s dynamic exclusion. The electrospray voltage applied was 2.0 kV. Automatic gain control (AGC) was used to prevent overfilling of the orbitrap; 5E4 ions were accumulated for generation of MS/MS spectra. For MS scans, the *m*/*z* scan range was 350 to 1800. Fixed first mass was set as 100 *m*/*z*.

The resulting MS/MS data was processed using MaxQuant with integrated Andromeda search engine (v.1.5.2.8, Martinsried, Germany). Tandem mass spectra were searched against Uniprot *Oryza sativa* database concatenated with reverse decoy database. Trypsin/P was specified as cleavage enzyme allowing up to 2 missing cleavages, 5 modifications per peptide. Mass error was set to 20 ppm for precursor ions and 0.02 Da for fragment ions. Carbamidomethylation on Cys was specified as fixed modification and oxidation on Met and acetylation on protein N-terminal were specified as variable modifications. False discovery rate (FDR) thresholds for protein, peptide and modification site were specified at 1%. Minimum peptide length was set at 7. For quantification method, iTRAQ-8plex was selected.

### 3.5. Bioinformatics Methods

The proteins were classified into three categories, biological process, cellular compartment and molecular function, by gene ontology annotation derived from the UniProt-GOA database (www.http://www.ebi.ac.uk/GOA/). Kyoto Encyclopedia of Genes and Genomes (KEGG) database was used to annotate protein pathway. The two-tailed Fisher’s exact test was employed to test the enrichment of the differentially expressed protein against all identified proteins. Correction for multiple hypothesis testing was carried out using standard false discovery rate control methods. The GO or pathway with a corrected *p*-value < 0.05 is considered significant.

## 4. Conclusions

Rice production is negatively affected by high temperature [5], with anthesis being the most sensitive stage [6,7], during which, high temperature usually occurs. Previously, a rice cultivar named N22 was identified as a HS tolerant germplasm. In comparison with HS sensitive cultivar Mianhui101, N22 showed much less spikelet sterility when they were exposed to HS. Comparative proteomics study on the anthers from both HS tolerant and sensitive cultivars was conducted in parallel showed that there are obvious differences between the two cultivars on the differentially expressed proteins. Many ribosomal proteins were specifically down-regulated in the HS sensitive cultivars, which indicates that injury on protein biosynthetic machinery ribosome in anther might be the main reason of spikelet fertility reduction in Mianhui101. Furthermore, specifically, increases of sHSPs, β-expansins and lipid transfer proteins in the resistant cultivar indicate these proteins might contribute to the high tolerance to HS in N22 cultivar. Exploring how these proteins function during HS, especially the linkage between these sHSPs and the stability of ribosome, will be very helpful in uncovering the mechanisms of HS resistance in rice.

## Figures and Tables

**Table 1:** N22-specific differentially expressed proteins.

Protein Accession	Protein Description	N22-35 °C/ N22-27 °C	N22-37 °C/ N22-27 °C	Functional Category
Up-regulated				
A2WLG6	Similar to 17.5 kDa class II heat shock protein	1.58 ± 0.4	2.74 ± 0.72	Stress response
A2X9T6	Similar to Low molecular weight heat shock protein precursor (mitochondrial small heat shock protein 22)	2.26 ± 0.48	2.93 ± 0.45	Stress response
B8ARU4	Peroxidase	1.53 ± 0.14	1.9 ± 0.27	Redox
Down-regulated				
A2WUN3	Pollen Ole e 1 allergen and extensin domain containing protein	0.46 ± 0.07	0.49 ± 0.05	Growth and development
A2XHE1	Aldose 1-epimerase	0.53 ± 0.06	0.4 ± 0.04	Metabolic
A2XNH0	α-expansin OsEXPA7	0.65 ± 0.11	0.36 ± 0.003	Cell wall
A2YNG3	Similar to glycerophosphoryl diester phosphodiesterase	0.45 ± 0.03	0.31 ± 0.04	Metabolic
A2ZDK1	Similar to β- d-xylosidase	0.66 ± 0.02	0.66 ± 0.02	Metabolic
A2ZFQ4	Protease inhibitor, lipid transfer protein (LTP), Postmeiotic anther development	0.52 ± 0.1	0.45 ± 0.05	Growth and development
B8ARP2	Similar to thaumatin-like protein	0.45 ± 0.08	0.44 ± 0.06	Chloroplast
B8B4J9	Similar to subtilisin-like protease	0.6 ± 0.03	0.49 ± 0.03	Metabolic
B8BB22	Exostosin-like family protein	0.62 ± 0.09	0.63 ± 0.15	Membrane part
B8BD35	Similar to Ervatamin B (EC 3.4.22.-) (ERV-B)	0.65 ± 0.09	0.57 ± 0.10	Metabolic
Q40629	Anther-specific protein RTS	0.54 ± 0.13	0.53 ± 0.11	Growth and development

**Table 2:** Mianhui101-specific differentially expressed proteins.

Protein Accession	Protein Description	101-35 °C/ 101-27 °C	101-37 °C/ 101-27 °C	Functional Category
Up-regulated				
A2WMG6	Salt stress root protein RS1	1.65 ± 0.24	1.51 ± 0.34	Stress response
A2XCH7	Tonoplast intrinsic protein (Tonoplast water channel)	1.62 ± 0.16	1.58 ± 0.20	transporter
A2Y1Q8	Similar to TCH2 (TOUCH 2); calcium ion binding	1.62 ± 0.15	1.78 ± 0.20	Signaling
A2Y8H4	Aldo/keto reductase domain containing protein	1.54 ± 0.06	1.59 ± 0.13	Metabolic
A2YCB4	Thioredoxin fold domain containing protein	1.57 ± 0.09	1.78 ± 0.19	Redox
A2YI28	Reticulon-like protein	1.59 ± 0.26	2.02 ± 0.20	Endoplasmic reticulum, ER
A2YM28	Thiamine thiazole synthase, chloroplastic	1.55 ± 0.12	1.8 ± 0.31	Starch metabolic
A2YQ88	Similar to PWWP domain containing protein	1.51 ± 0.22	1.51 ± 0.29	DNA binding
A2Z0W7	Mannose-6-phosphate isomerase	1.81 ± 0.07	1.89 ± 0.20	Metabolic
B8B8G2	Tubulin alpha chain	1.54 ± 0.24	1.52 ± 0.23	Cell structure
B8BF32	HAD superfamily hydrolase-like, type 3 domain containing protein	2.03 ± 0.59	3.15 ± 0.60	Metabolic
Down-regulated				
A2X3J5	40S ribosomal protein S3a	0.66 ± 0.17	0.63 ± 0.12	Ribosome
A2X6N1	60S ribosomal protein L6	0.6 ± 0.02	0.61 ± 0.05	Ribosome
A2XCC4	Similar to 60S ribosomal protein L21	0.46 ± 0.07	0.56 ± 0.07	Ribosome
A2XM46	Similar to 60S ribosomal protein L13a-4	0.65 ± 0.05	0.62 ± 0.04	Ribosome
A2YIS2	Similar to 60S ribosomal protein L4	0.65 ± 0.07	0.66 ± 0.06	Ribosome
A2YNM6	Similar to 60S ribosomal protein L27a-3	0.59 ± 0.07	0.66 ± 0.05	Ribosome
A2YVK0	Similar to 60S ribosomal protein L34	0.6 ± 0.14	0.55 ± 0.10	Ribosome
A2ZLS7	Similar to 60S ribosomal protein L2 (fragment)	0.61 ± 0.05	0.59 ± 0.05	Ribosome
B8AHZ6	40S ribosomal protein S8	0.62 ± 0.03	0.63 ± 0.03	Ribosome
B8B6Q3	Similar to Pleiotropic drug resistance protein 3	0.66 ± 0.01	0.56 ± 0.01	Transporter

## Supplementary Materials

Supplementary materials can be found at www.mdpi.com/1422-0067/18/9/1811/s1.
