# Supplementary material for: iTRAQ-Based Quantitative Proteomics Analysis on Rice Anther Responding to High Temperature
Source: Int J Mol Sci. 2017 Aug 23;18(9):1811. doi: 10.3390/ijms18091811 (PMC5618475; doi:10.3390/ijms18091811)
Supplement: Supplementary file 1 [file ijms-18-01811-s001.zip › Table S1 Temperature setting of the growth chamber for different heat treatment.pdf]

Table S1 Temperature setting of the growth chamber for different heat treatment. The second line of the table head indicate different time of a day. The last four lines indicate the heat treatments and corresponding temperatures set at each time point (^{o}C).

**Heat treatment**	**Time point**																							
	21:00	22:00	23:00	0:00	1:00	2:00	3:00	4:00	5:00	6:00	7:00	8:00	9:00	10:00	11:00	12:00	13:00	14:00	15:00	16:00	17:00	18:00	19:00	20:00
CK	28	28	27	26	26	25	25	25	25	25	26	26	27	28	29	30	30	31	31	30	30	29	29	28
33 ^{o}C	30	30	29	29	28	28	27	27	27	27	28	28	30	30	32	32	33	33	32	32	31	31	30	30
35 ^{o}C	32	31	31	30	30	29	29	29	29	29	29	30	31	32	33	34	35	35	35	35	35	34	34	32
37 ^{o}C	34	33	33	32	32	32	31	31	31	31	31	32	32	34	34	36	36	37	37	37	37	36	36	34
